# A Manual of Otology

**Published:** 1907-03

**Authors:** 


**A Manual of Otology.** By **Gorham Bacon,** A.B., M.D., Professor of Otology in the College of Physicians and Surgeons, New York. With an Introductory Chapter by Clarence John Blake, M.D., Professor of Otology in Harvard University. Fourth Edition. 12mo., pp. 485. With 134 Illustrations and 11 Plates. New York and Philadelphia: Lea Brothers & Co. 1906. (Price, $2.25).

When a book, crowned with success, gallops through three editions in a short time and a fourth revision is necessary by the demand of the profession, it goes without saying that there is more than good publishing behind it. Bacon's Manual of Otology in the fourth edition has been improved over its predecessors in more than the mere rewriting of its preface. The author has brought it up to date and kept pace with all the advances made in otologic work since the third edition was placed before the profession. New illustrations have been added and newer topics considered, among them osteomyletis and primary jugular bulb thrombosis and suppurative inflammation of the labyrinth. Those chapters dealing with leukocytosis, lumbar puncture and the treatment of facial paralysis have been rewritten. A very laudable addition is the appendix wherein is considered the method of preparing smears of pus, the making of cultivations and the physiologic inoculation experiments. In its present form the book is one of the most serviceable of its field and will be of inestimable value to the general practitioner.

